# Karyotypic diversity and evolutionary trends in the Neotropical catfish genus *Hypostomus* Lacépède, 1803 (Teleostei, Siluriformes, Loricariidae)

**DOI:** 10.3897/CompCytogen.v6i4.4028

**Published:** 2012-12-15

**Authors:** Anderson Luis Alves, Rafael Splendore de Borba, Claudio Oliveira, Mauro Nirchio, Angel Granado, Fausto Foresti

**Affiliations:** 1 Embrapa Pesca e Aquicultura (CNPASA), Palmas, Tocantins, Brazil; 2 Laboratório de Citogenética, Univ Estadual Paulista “Julio de Mesquita Filho” - UNESP, Rio Claro, São Paulo State, Brazil; 3 Departamento de Morfologia, Instituto de Biociências, Univ Estadual Paulista Julio de Mesquita Filho, Botucatu, SP, Brasil; 4 Instituto Limnológico, Universidad de Oriente, Caicara del Orinoco, Estado Bolívar, Venezuela

**Keywords:** Armoured-catfish, Loricariidae, *Hypostomus*, karyotype evolution, Ag-NORs, centric fission, polyploidy

## Abstract

The family Loricariidae with 813 nominal species is one of the largest fish families of the world. Hypostominae, its more complex subfamily, was recently divided into five tribes. The tribe Hypostomini is composed of a single genus, *Hypostomus* Lacépède, 1803, which exhibits the largest karyotypic diversity in the family Loricariidae. With the main objective of contributing to a better understanding of the relationship and the patterns of evolution among the karyotypes of *Hypostomus* species, cytogenetic studies were conducted in six species of the genus from Brazil and Venezuela. The results show a great chromosome variety with diploid numbers ranging from 2n=68 to 2n=76, with a clear predominance of acrocentric chromosomes. The Ag-NORs are located in terminal position in all species analyzed. Three species have single Ag-NORs (*Hypostomus albopunctatus* (Regan, 1908), *Hypostomus* prope *plecostomus* (Linnaeus, 1758), and *Hypostomus* prope *paulinus* (Ihering, 1905)) and three have multiple Ag-NORs (*Hypostomus ancistroides* (Ihering, 1911), *Hypostomus* prope *iheringi* (Regan, 1908), and *Hypostomus strigaticeps* (Regan, 1908)). In the process of karyotype evolution of the group, the main type of chromosome rearrangements was possibly centric fissions, which may have been facilitated by the putative tetraploid origin of *Hypostomus* species. The relationship between the karyotype changes and the evolution in the genus is discussed.

## Introduction

**Figure 1.**
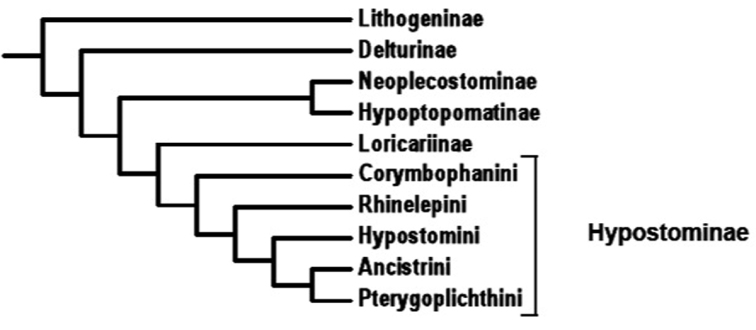
Phylogeny of the family Loricariidae proposed by Armbruster (2004).

The subfamily Hypostominae with about 386 species (Reis et al. 2006) is the largest one in the family Loricariidae. The subfamily Hypostominae can only be recognized as monophyletic with the inclusion of the old subfamily Ancistrinae and the exclusion of some genera more related to the subfamily Neoplecostominae (Armbruster 2004). This subfamily is divided into five tribes: Corymbophanini, Rhinelepini, Hypostomini, Ancistrini, and Pterygoplichithini (Armbruster 2004) (Fig. 1). The tribe Hypostomini, with the only genus *Hypostomus*, has the greatest number of Hypostominae species (Reis et al. 2003).

The genus *Hypostomus* is the most representative in the family (Weber 2003, Hollanda Carvalho et al. 2010) with 126 species distributed from Central America to southern South America (Zawadzki et al. 2010). Species of the genus display phenotypic plasticity that makes difficult to obtain diagnostic characters for the group (Armbruster 2004).

Recent studies suggested that the genus *Hypostomus* might be composed of some monophyletic groups (Muller and Weber 1992, Montoya-Burgos 2003, Armbruster 2004, Zawadzki et al. 2004, Alves et al. 2006). This suggestion is confirmed by extensive morphological variation in the genus combined with a largest variety of diploid numbers and karyotype formulae in Loricariidae (Artoni and Bertollo 1996, Alves et al. 2006), with diploid numbers ranging from 2n=52 in *Hypostomus emarginatus* (Valenciennes, 1840) (Artoni 1996) to 2n=84 in *Hypostomus* sp. 2 (Cereali et al. 2008) (Table 1).

**Table 1.** A summary of the cytogenetic data available for the genus *Hypostomus*. 2n = diploid number; M = metacentric; SM = submetacentric; ST = subtelocentric; A = acrocentric.

**Species**	**Locality**	**2n**	**Karyotypic formulae**	**References**
*Hypostomus affinis* (Steindachner, 1877)	Paraitinga River, São Paulo, Brazil	66	14M, 14SM, 12ST, 26A	Kavalco et al. (2004)
	Jacuí stream (SP)	66	14M, 14SM, 12ST, 26A	Fenerich et al. (2004)
*Hypostomus albopunctalus* (Regan, 1908)	Mogi-Guaçu River, São Paulo, Brazil	74	10M, 20SM, 44ST/A	Artoni and Bertollo (1996)
	Corumbataí River, São Paulo, Brazil	74	10M, 20M, 16ST, 28A	Present study
*Hypostomus ancistroides* (Ihering, 1911)	--	68	10M, 28SM, 30ST/A	Michelle et al. (1977)
	Araquá River, São Paulo, Brazil	68	18M, 10SM, 12ST, 28A	Alves et al. (2006)
	Corumbataí River, São Paulo, Brazil	68	16M, 4SM, 16ST, 32A	Present study
	Mogi-Guaçu River, São Paulo, Brazil	68	16M, 18SM, 34ST/A	Artoni and Bertollo (1996)
	Paranapanema River, São Paulo, Brazil	68	10M, 26SM, 32ST/A	Rubert et al. (2011)
*Hypostomus* prope *auroguttatus* (Kner, 1854)	Mogi-Guaçu River, São Paulo, Brazil	76	8M, 30SM, 38ST/A	Artoni and Bertollo (1996)
*Hypostomus cochliodon* (Kner, 1854)	Salobra river and Salobrinha stream (MS)	64	16M, 20SM, 28ST-A (male)/ 16M, 19SM, 27ST-A (female)	Cereali (2006)
*Hypostomus emarginatus* (Valenciennes, 1840)	Araguaia River, Mato Grosso, Brazil	52	16M, 30SM, 6ST	Artoni (1996)
*Hypostomus goyazensis* (Regan, 1908)	Vermelho River, Goiás, Brazil	72	10M, 16SM, 10ST, 36A	Alves et al. (2006)
*Hypostomus* prope *iheringi* (Regan, 1908)	Corumbataí River, São Paulo, Brazil	74	10M, 14M, 20ST, 30A	Present study
*Hypostomus macrops* (Eigenmann & Eigenmann, 1888)	--	68	10M, 14SM, 44ST/A	Michelle et al. (1977)
*Hypostomus nigromaculatus* (Schubart, 1964)	Tibagi River, Paraná, Brazil.	76	6M, 20SM, 50ST/A	Rubert et al. (2008)
	Mogi-Guaçu River, São Paulo, Brazil	76	8M, 20SM, 48ST/A	Rubert et al. (2008)
*Hypostomus paulinus* (Ihering, 1905)	--	74	10M, 20SM, 44ST/A	Michelle et al. (1977)
*Hypostomus* prope *paulinus* (Ihering, 1905)	Corumbataí River, São Paulo, Brazil	76	6M, 18M, 12ST, 40A	Present study
*Hypostomus* prope *paulinus* (Ihering, 1905)	Corumbataí River, São Paulo, Brazil	76	6M, 18M, 12ST, 40A	Present study
*Hypostomus plecostomus* (Linnaeus, 1758)	--	54	24M/SM, 12ST, 18A	Muramoto et al. (1968)
*Hypostomus* prope *plecostomus* (Linnaeus, 1758)	Orinoco River, Bolivar, Venezuela	68	12M, 16M, 12ST, 28A	Present study
*Hypostomus regani* (Ihering, 1905)	Mogi-Guaçu River, São Paulo, Brazil	72	10M, 20SM, 42ST/A	Artoni and Bertollo (1996)
	Paranapanema River, São Paulo, Brazil	72	10M, 18SM, 44ST/A	Rubert et al. 2011
	Araguá River, São Paulo, Brazil	72	12M, 18SM, 26ST, 16A	Alves et al. (2006)
*Hypostomus strigaticeps* (Regan, 1908)	Corumbataí River, São Paulo, Brazil	74	10M, 14M, 14ST, 36A	Present study
	Mogi-Guaçu River, São Paulo, Brazil	74	8M, 4SM, 62ST/A	Michelle et al. (1977)
	Paranapanema River, São Paulo, Brazil	72	10M, 16SM, 46ST/A	Rubert et al. (2011)
*Hypostomus* sp. 2	Salobrinha stream, Mato Grosso do Sul, Brazil	84	6M, 16SM, 62ST/A	Cereali et al. (2008)
*Hypostomus* sp. 3	Perdido River, Mato Grosso do Sul, Brazil	82–84	6M, 16SM, 64ST/A - 6M, 12SM, 66ST/A	Cereali et al. (2008)
*Hypostomus* sp. A	Rincão Stream, São Paulo, Brazil	70	18M, 14SM, 38ST/A	Artoni and Bertollo (1996)
*Hypostomus* sp. B	Mogi-Guaçu River, São Paulo, Brazil	72	12M, 18SM, 42ST/A	Artoni and Bertollo (1996)
*Hypostomus* sp. C	Mogi-Guaçu River, São Paulo, Brazil	68	10M, 18SM, 40ST/A	Artoni and Bertollo (1996)
*Hypostomus* sp. D1	Mogi-Guaçu River, São Paulo, Brazil	72	10M, 26SM, 36ST/A	Artoni and Bertollo (1996)
*Hypostomus* sp. D2	Mogi-Guaçu River, São Paulo, Brazil	72	14M, 20SM, 38ST/A	Artoni and Bertollo (1996)
*Hypostomus* sp. E	Mogi-Guaçu River, São Paulo, Brazil	80	8M, 16SM, 56ST/A	Artoni and Bertollo (1996)
*Hypostomus* sp. F	São Francisco River, Minas Gerais, Brazil	76	10M, 16SM, 50ST/A	Artoni (1996)
*Hypostomus* sp. G	Araguaia River, Mato Grosso, Brazil	64	14M, 24SM, 26ST/A	Artoni (1996)
*Hypostomus* sp. Xingu-1	Xingu River, Pará, Brazil	64	32M/SM, 32ST/A	Milhomem et al. (2010)
*Hypostomus* sp. Xingu-2	Xingu River, Pará, Brazil	66	32M/SM, 34ST/A	Milhomem et al. (2010)
*Hypostomus* sp. Xingu-3	Xingu River, Pará, Brazil	65	38M/SM, 26ST/A, 1b	Milhomem et al. (2010)

Cytogenetic studies in *Hypostomus* are relatively well documented (Table 1). In a review of genus cytogenetic data by Bueno et al. (2011) the relations between diploid number and karyotypic formulae of genus were established. However, several problems were not yet solved, including the pattern of karyotype evolution in Hypostomini. In the present study, six species of *Hypostomus* were karyotyped and the results employed to discuss the karyotype evolution of the genus.

## Material and methods

Cytogenetic analyses were performed on chromosomal preparations obtained from six species. Five species were collected in the Corumbataí River, São Paulo, Brazil: *Hypostomus ancistroides* (Ihering, 1911) (6 males and 4 females) (LBP 2544), *Hypostomus albopunctatus* (Regan, 1908) (5 males and 6 females) (LBP 2547), *Hypostomus strigaticeps* (Regan, 1908) (6 males and 7 females) (LBP 2545), *Hypostomus* prope *iheringi* (Regan, 1908) (5 males and 4 females) (LBP 1674), and *Hypostomus* prope *paulinus* (Ihering, 1905) (5 males and 6 females) (LBP 2548). One species of *Hypostomus* prope *plecostomus* (Linnaeus, 1758) (3 males and 2 females) (LBP 2198) was collected in the Orinoco River, Bolivar, Venezuela. Vouchers were deposited in the fish collection of Laboratório de Biologia e Genética de Peixes (LBP), UNESP, Botucatu, São Paulo, Brazil.

Chromosome preparations were obtained from kidney tissues using the technique described by Foresti et al. (1993). Silver staining of the nucleolus organizer regions (Ag-NORs) was performed according to the technique proposed by Howell and Black (1980). Chromosome morphology was determined on the basis of arm ratio, as proposed by Levan et al. (1964) and the chromosomes were classified as metacentrics (M), submetacentrics (SM), subtelocentrics (ST) and acrocentrics (A).

## Results and discussion

**Figure 2.**
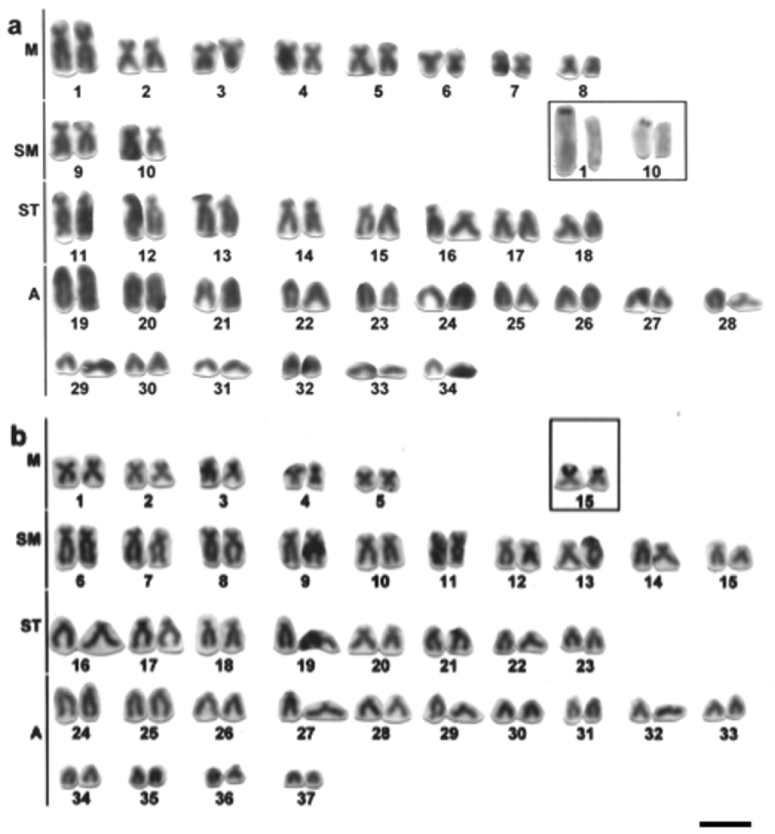
Giemsa stained karyotypes of *Hypostomus* **a** *Hypostomus ancistroides*, 2n=68 **b** *Hypostomus albopunctatus*, 2n=74. Ag-NOR-bearing chromosome pairs in the insets. Bar = 10µm.

*Hypostomus ancistroides* has karyotype with 2n=68 (16M, 4SM, 16ST, 32A) and terminal Ag-NORs on the short arm of the chromosome pair 1 (M) and pair 10 (SM) (Fig. 2a).

*Hypostomus albopunctatus* has 2n=74 (10M, 20SM, 16ST, 28A) and terminal Ag-NORs on the short arm of the chromosome pair 15 (SM) (Fig. 2b).

**Figure 3.**
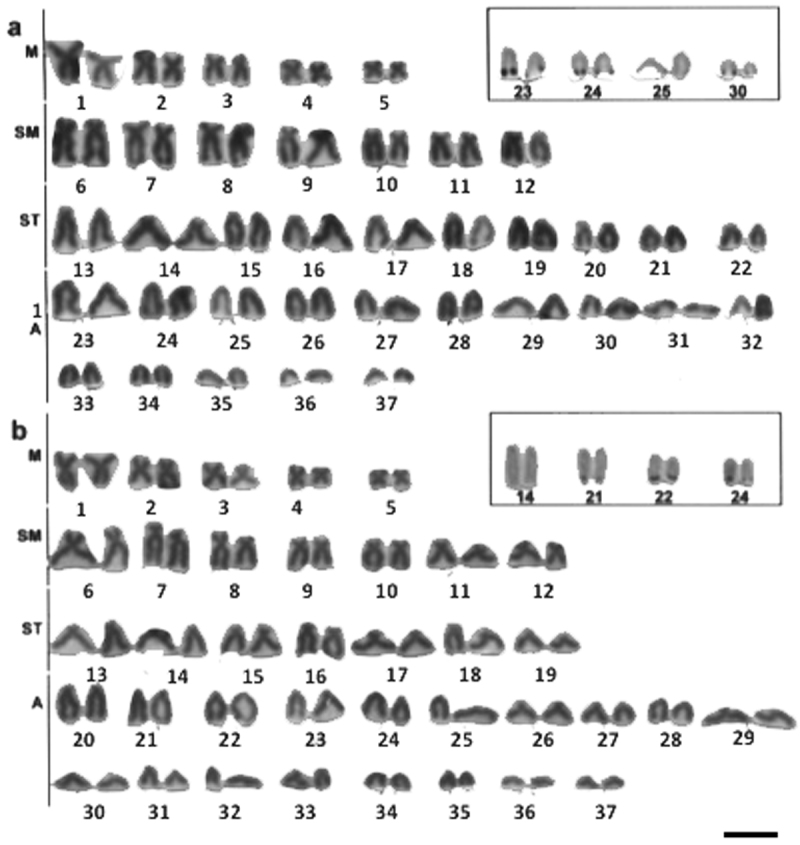
Giemsa stained karyotypes of *Hypostomus* **a** *Hypostomus* prope *iheringi*, 2n=74 **b** *Hypostomus strigaticeps*, 2n=74. Ag-NOR-bearing chromosome pairs in the insets. Bar = 10µm.

*Hypostomus*prope *iheringi* has 2n=74 (10M, 14SM, 20ST, 30A) and terminal Ag-NORs on the long arms of the chromosome pairs 23, 24, 25, 30 (A) (Fig. 3a).

**Figure 4.**
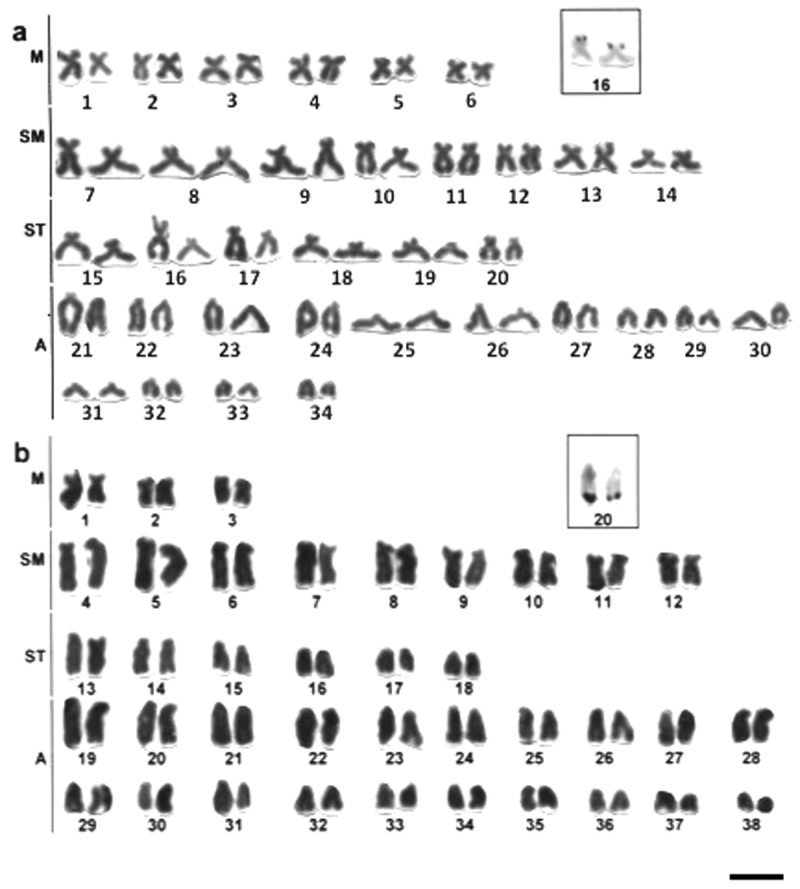
Giemsa stained karyotypes of *Hypostomus* **a** *Hypostomus* prope *plecostomus*, 2n=68 **b** *Hypostomus* prope *paulinus*, 2n=76. Ag-NOR-bearing chromosome pairs in the insets. Bar = 10µm.

*Hypostomus* prope *paulinus* has 2n=76 (6M, 18SM, 12ST, 40A) and terminal Ag-NORs on the long arm of the chromosome pair 20 (A) (Fig. 4b).

*Hypostomus* prope *plecostomus* has 2n=68 (12M, 16SM, 12ST, 28A) and terminal Ag-NORs on the short arm of the chromosome pair 16 (ST) (Fig. 4a).

*Hypostomus strigaticeps* has 2n=74 (10M, 14SM, 14ST, 36A) and terminal Ag-NORs on the short arm of the chromosome pair 14 (ST) and on the long arm of the chromosome pairs 21, 22 e 24 (A) (Fig. 3b).

The genus *Hypostomus* seems to be the karyotypically most derived genus in Loricariidae (Rubert et al. 2011), the variation of diploid number observed in the six species of *Hypostomus* analyzed (2n=68 to 2n=76) confirms this hypothesis. All species analyzed exhibited a large number of acrocentric chromosomes, reinforcing the hypothesis that higher diploid numbers are positively related to higher number of acrocentric chromosomes in *Hypostomus* (Artoni and Bertollo 2001). According to Oliveira and Gosztonyi (2000), high diploid numbers may represent a derived characteristic in siluriforms.

Three species had single Ag-NORs (*Hypostomus albopunctatus*, *Hypostomus* prope *plecostomus*, and *Hypostomus* prope *paulinus*); and the three others had multiple Ag-NORs (*Hypostomus ancistroides*, *Hypostomus* prope *iheringi*, and *Hypostomus strigaticeps*). All species presented terminal Ag-NORs, a marked characteristic of the species of this genus. The occurrence of multiple Ag-NORs is the most common characteristic among the Hypostomini, however, this phenotype is considered a derived characteristic among siluriforms (Oliveira and Gosztonyi 2000), which usually predominate single Ag-NORs.

Differences in the karyotype formulae or in the number and position of Ag-NORs are common in species that do not present extensive migration behaviour, since isolated populations are more commonly involved in inbreeding processes, which makes the fixation of chromosome rearrangements easier (Almeida-Toledo et al. 2000). This kind of phenomenon has been extensively documented in fishes as in *Astyanax scabripinnis* (Jenyns, 1842) (Moreira-Filho and Bertollo 1991, Maistro et al. 1998, Alves and Martins-Santos 2002). On the other hand one of the most important problems associated with the study of the genus *Hypostomus* is the correct species identification due to the large number of species as well as the close morphological similarity among species (Armbruster 2004). Thus, Table 1 shows many samples identified as *Hypostomus* sp., which reflects our poor taxonomic knowledge of the group. Among the *Hypostomus* species, the high diploid number is coincident with a high the number of uniarmed chromosomes (Table 1), suggesting the occurrence of a large number of centric fissions in the karyotypic evolution of the group (Artoni and Bertollo 1996). This hypothesis is reinforced considering that the species of Rhinelepini, the sister group of Hypostomini, has 2n=54 chromosomes (Alves et al. 2003, Alves et al. 2005, Alves et al. 2006). The occurrence of a polyploidy event in the origin of the tribe Hypostomini may explain the existence of duplicated centromeres and telomeres that could have been activated in the centric fissions rearrangements.

Thus, in the ancestor of Hypostomini an extensive process of chromosome fusions should have occurred changing a putative original karyotype with 2n=108 chromosomes into a karyotype with 2n=54 chromosomes. The alternative hypothesis that species of *Hypostomus* with high diploid numbers are the most primitive, suggesting that new chromosome fusions are reducing the diploid numbers in the genus, is not corroborated by the phylogenies available for the genus (Montoya-Burgos 2003, Armbruster 2004). Considering that the available phylogenies for the genus Hypostomus are very limited regarding the number of species and precise fish identification, further phylogenetic studies including karyotyped fishes are fundamental for a better understanding of the chromosome evolution in *Hypostomus*.
